# Data-Driven Clustering of Plantar Thermal Patterns in Healthy Individuals: An Insole-Based Approach to Foot Health Monitoring

**DOI:** 10.3390/bioengineering12020143

**Published:** 2025-02-01

**Authors:** Mark Borg, Stephen Mizzi, Robert Farrugia, Tiziana Mifsud, Anabelle Mizzi, Josef Bajada, Owen Falzon

**Affiliations:** 1 Centre for Biomedical Cybernetics, University of Malta, MSD 2080 Msida, Malta; owen.falzon@um.edu.mt; 2 Department of Podiatry, Faculty of Health Sciences, University of Malta, MSD 2080 Msida, Malta; stephen.mizzi@um.edu.mt (S.M.); robert.farrugia@um.edu.mt (R.F.); tiziana.mifsud.06@um.edu.mt (T.M.); anabelle.mizzi@um.edu.mt (A.M.); 3 Tarsos, ZBR 1061 Zabbar, Malta; 4 Department of AI, Faculty of ICT, University of Malta, MSD 2080 Msida, Malta; josef.bajada@um.edu.mt

**Keywords:** wearable sensors, smart insoles, foot health monitoring, plantar thermal patterns, thermal maps, data-driven clustering

## Abstract

Monitoring plantar foot temperatures is essential for assessing foot health, particularly in individuals with diabetes at increased risk of complications. Traditional thermographic imaging measures foot temperatures in unshod individuals lying down, which may not reflect thermal characteristics of feet in shod, active, real-world conditions. These controlled settings limit understanding of dynamic foot temperatures during daily activities. Recent advancements in wearable technology, such as insole-based sensors, overcome these limitations by enabling continuous temperature monitoring. This study leverages a data-driven clustering approach, independent of pre-selected foot regions or models like the angiosome concept, to explore normative thermal patterns in shod feet with insole-based sensors. Data were collected from 27 healthy participants using insoles embedded with 21 temperature sensors. The data were analysed using clustering algorithms, including k-means, fuzzy c-means, OPTICS, and hierarchical clustering. The clustering algorithms showed a high degree of similarity, with variations primarily influenced by clustering granularity. Six primary thermal patterns were identified, with the “butterfly pattern” (elevated medial arch temperatures) predominant, representing 51.5% of the dataset, aligning with findings in thermographic studies. Other patterns, like the “medial arch + metatarsal area” pattern, were also observed, highlighting diverse yet consistent thermal distributions. This study shows that while normative thermal patterns observed in thermographic imaging are reflected in insole data, the temperature distribution within the shoe may better represent foot behaviour during everyday activities, particularly when enclosed in a shoe. Unlike thermal imaging, the proposed in-shoe system offers the potential to capture dynamic thermal variations during ambulatory activities, enabling richer insights into foot health in real-world conditions.

## 1. Introduction

Monitoring plantar foot temperatures provides crucial insights into foot health, particularly for individuals at high risk of complications. In diabetic patients, for example, measuring and analysing plantar surface temperatures has proven to be a potentially effective method in the early detection and prediction of ulceration risk, enabling timely intervention to prevent severe outcomes [1,2].

Infrared thermography is a well-established technique for capturing plantar surface temperatures, offering advantages such as high accuracy and a non-invasive approach [3,4,5,6,7,8,9,10,11]. Despite these benefits, the method has limitations: it requires participants to be barefoot, remain stationary (typically in a supine position) during imaging, and is generally conducted in a controlled clinical setting.

Recent advances in wearable sensor technologies (including miniaturization, flexible, and stretchable electronics, combined with improvements in reliability and accuracy) have opened up the possibility of using in-shoe temperature acquisition systems [12,13,14,15]. In particular, smart insoles equipped with an ever-increasing number of temperature sensors, can be used to acquire thermal maps of the plantar surface [16,17,18,19,20]. One advantage of such smart insoles is their ability to monitor foot temperatures continuously, even during motion and in uncontrolled real-world environments. This represents a significant improvement over traditional thermography, which typically requires a static posture and a controlled clinical setting for accurate measurement.

### 1.1. Related Work

**Figure 1:**
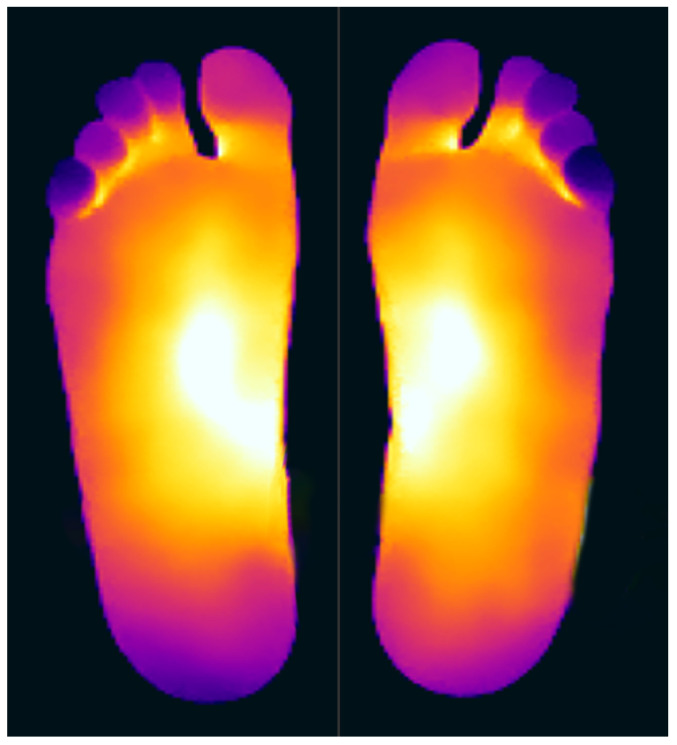
Thermogram showing the Butterfly pattern.

When analysing the thermal distribution patterns of the plantar surface, research consistently highlights distinct differences in these patterns between those belonging to healthy feet and those affected by conditions such as peripheral artery disease (PAD) or diabetic foot ulceration [21]. Healthy individuals at rest, typically exhibit a ‘symmetric bilateral butterfly’ pattern, characterized by elevated temperatures in the medial arches [22,23,24,25]—see Figure 1 for an example. Although this thermal pattern is prevalent in the healthy population and has been observed in approximately half of the cases across studies (56% [22], 46.9% [24], 44% [25], 47.2% [26]), it is by no means the only thermal pattern observed.

In the pioneering study by Nagase et al. [24], thermographic patterns were manually classified for 161 participants, 32 of which were non-diabetic control subjects. Their method divided the plantar surface into four regions based on the angiosome concept, with binary labels indicating whether a region exhibited higher temperatures than the rest. In the case of the control group, they found that their thermal patterns fall into seven different categories, with 46.9% belonging to the butterfly pattern class (the largest category). Notably, however, 25% of the control subjects exhibited thermal patterns that did not align with any of the predefined categories; termed ‘atypical’ by Nagase et al. This classification, conducted manually, was noted as potentially too complex for regular clinical application and prone to subjectivity.

Mori et al. [25] addressed the limitations of Nagase’s approach [24] by implementing an automated classification system, using a mean shift algorithm for region-based segmentation, followed by spatial clustering of the image regions. Although this approach automated much of the process, they maintained Nagase’s use of angiosomes for the foot regions and the use of binary labels to describe temperature variations. Mori’s system classified the thermal patterns of the forefoot of control subjects into four categories—three of which aligned with Nagase’s findings, while the fourth represented a newly identified category. Nonetheless, as with Nagase et al. [24], 27% of control subjects were classified as anomalous, exhibiting thermal patterns not matching any of the predefined categories.

Hernandez-Contreras et al. [7] departed from the binary labelling used for describing temperature differences in previous studies. They developed a quantitative approach to automatically classify thermal patterns by calculating deviations from a reference butterfly pattern in healthy controls. They introduced a multi-threshold index to group abnormal patterns into five risk categories, with higher thresholds indicating greater risk. However, this approach treated all controls as a single group, despite evidence of differences in their thermal patterns, and the thresholds were manually chosen.

In later work, Hernandez-Contreras et al. [27] applied clustering on the mean foot temperatures across predefined foot regions, following the fitting of a beta mixture model to the temperature distribution along the principal plantar axis. Focusing on four specific regions, with particular emphasis on differences between the toes and heel areas, they utilized the k-means algorithm as a proof of concept, resulting in three distinct clusters. Like their previous work, they treated the control group as a single class, and their analysis relied on pre-segmented regions and predefined cluster counts.

All these studies used thermographic data to classify thermal foot patterns. To the best of our knowledge, no attempts have classified normative foot thermal patterns using temperature data from wearable insole-based sensors, nor has fully automated clustering been explored without relying on predefined foot regions, such as those corresponding to the angiosome model.

### 1.2. Objectives

The aim of this study is to establish what normative (healthy) thermal patterns exist in shod feet. Furthermore, these patterns are compared with the established normative temperature patterns reported in the literature for unshod feet.

More specifically, an insole-based temperature measurement system was employed to analyse the thermal patterns exhibited when the foot is shod, which opens up a new approach to analysing temperature closer to real-life scenarios. These were then compared with thermal patterns obtained from traditional thermographic imaging of unshod feet. From this comparative investigation of foot temperature pattern classification in shod feet, this study assessed how effectively the classification outcomes reported in prior research on unshod feet using thermography translate to the context of shod feet monitored through wearable systems.

A purely data-driven clustering technique was used, free from assumptions or pre-segmentation of foot regions. Previous studies segmented the foot into anatomical regions aligned with angiosomes and used angiosome-level temperatures as the basis of data interpretation. In contrast, the new approach presented here leverages a denser and more granular representation of the plantar surface. By avoiding reliance on predefined models, our approach circumvented the risk of conditioning the clustering process on predefined anatomical regions.

For this preliminary study, temperature measurements were acquired from participants lying in a supine position. Additionally, this study does not seek to draw conclusions from the thermal patterns regarding the participants’ actual health conditions.

## 2. Methods

### 2.1. Experimental Setup

The data for this study was collected during a controlled observational study involving 27 healthy individuals without a history of significant medical, surgical, vascular, or neurological disease. Peripheral arterial perfusion was assessed by Doppler waveform analysis, ankle-brachial pressure index (ABPI) and toe-brachial index pressure (TBPI) by a clinician with over 10 years experience in the field. Recruited participants had triphasic waveforms, ABPI between 1 and 1.29 and TBPI > 0.7. Participants with a history of alcohol abuse or smoking were excluded. Demographic data were recorded for each participant: the mean age of the participants was 36.1 years (13.8 S.D.), with 20 and 74 years being the minimum and maximum age, respectively. A total of 80.8% of the participants were male, while 19.2% female.

**Figure 2:**
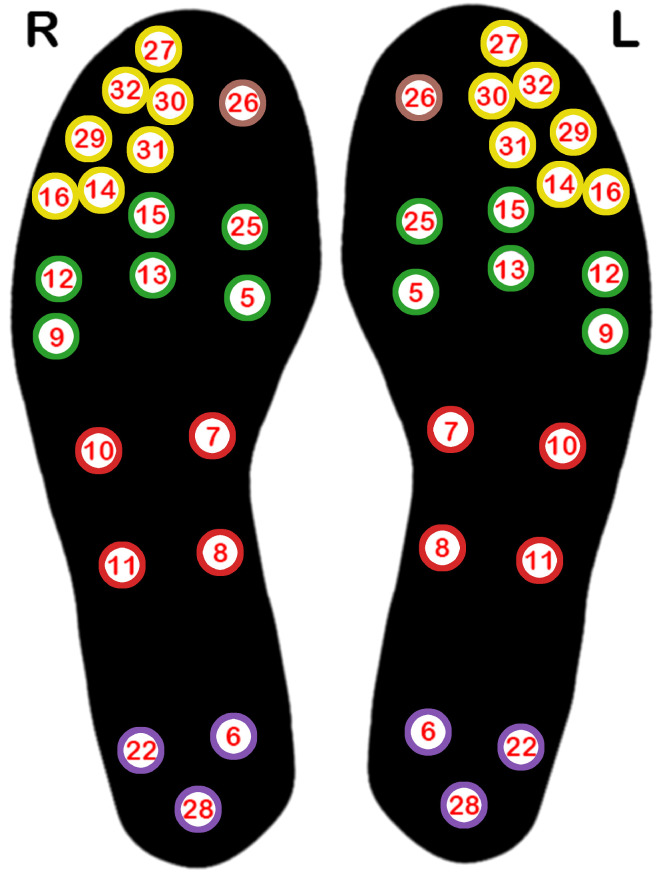
The position and the sensor number of the 21 temperature sensors embedded in each left and right insole. The colour indicates the area of the foot the sensor lies in: light brown: hallux; yellow: toe area; green: ball of the foot (metatarsal area); red: arch; purple: heel.

Temperature data were gathered using insoles equipped with 21 embedded thermistors, positioned as illustrated in Figure 2. These temperature sensors communicated wirelessly via Bluetooth, transmitting data to a mobile app at a sampling rate of 0.1 Hz. Both the insoles and mobile app were designed and developed by Tarsos [28], as part of an earlier phase of this project. Different insole sizes were used to accommodate different foot sizes.

During data capture, participants wore shoes with the smart insoles and lay in a supine position for 10 min. For this preliminary study, the participants’ pose was restricted to a supine position to establish a baseline of thermal patterns in shod feet, comparable to those obtained through traditional thermography of unshod feet. Future work will explore how thermal patterns vary in different static poses (sitting, standing) and ambulatory phases (walking) compared to the baseline patterns established by this study.

Thermographic images were taken before the feet were shod, prior to temperature data acquisition via the insoles, in order to check that the initial temperature distribution obtained from the in-shoe sensors was similar to that obtained from the unshod feet using thermal cameras. A FLIR E95 thermal camera with 464×348 spatial resolution was used for this purpose. Fifteen minutes were allotted for foot acclimatization before capturing thermograms.

This research was approved by the University of Malta Research Ethics Committee. Written informed consent was obtained from all of the participants. All research efforts conducted on humans were in compliance with the World Medical Association’s Declaration of Helsinki.

### 2.2. Data Preprocessing

**Figure 3:**
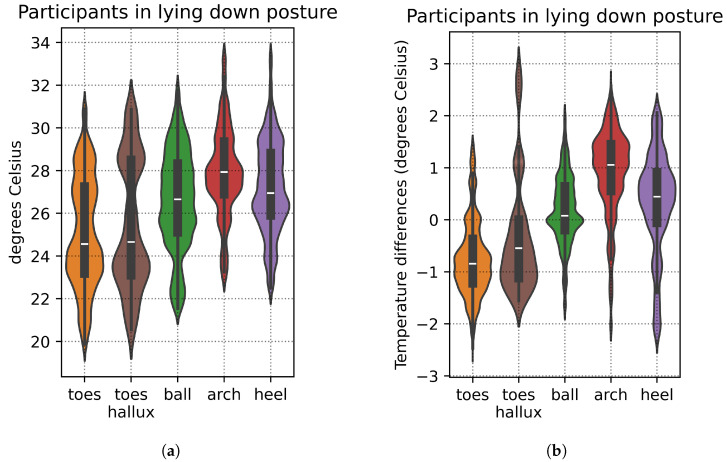
(**a**) Violin plots of the temperature value distributions of the participants when in a lying down posture with shod feet, grouped by foot region. (**b**) The distributions of the relative temperature differences after subtracting each participant’s temperature values from their mean foot temperature.

Despite the time allotted for foot acclimatization and all participants reaching thermal equilibrium in the lying down posture with shod feet, there was still some natural variation in foot temperatures at the start of the recording sessions, with certain individuals exhibiting warmer feet than others. Taking the mean foot temperature for each participant (computed as the mean temperature across all the 21 insole sensors of the participant’s foot), we then quantified the variation across all participants: mean 26.40°C, S.D. 2.13 °C. Figure 3a provides a breakdown of this variation in foot temperature by foot region.

To minimize this variation and account for potential bias from the participants’ initial temperatures, we focus on relative temperature differences instead: for each participant, the individual sensor values are adjusted by subtracting them from the mean temperature of that participant’s foot. This enables more consistent comparisons across participants. With relative temperature differences, the total variation across all participants is reduced: mean 0.00 °C, S.D. 0.19 °C. Figure 3b shows a breakdown of this variation by foot region, which is less than that shown in Figure 3a.

### 2.3. Thermal Map Generation

The thermal map is created by interpolating sensor data across the plantar surface of the foot, using a 2D interpolator based on the Clough–Tocher scheme [29]. This approach applies piecewise cubic interpolation over triangular elements, yielding smooth, continuous surfaces between points. Key advantages of this interpolator include its effectiveness with irregularly scattered data points, such as the sensor placements on our smart insoles, and its ability to capture fine detail through cubic interpolation, valuable for mapping subtle temperature variations in foot thermal patterns. A limitation is its sensitivity to outliers, which we mitigate through outlier removal and signal smoothing. Clough–Tocher interpolation has seen successful applications in medical imaging [30,31]. In our work, we utilize the Clough–Tocher implementation available in the SciPy library [32].

**Figure 4:**
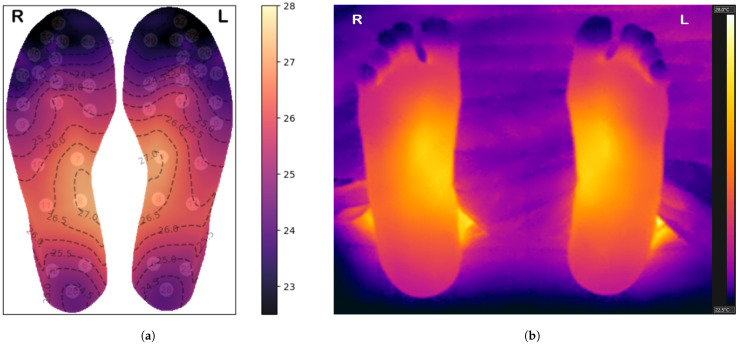
(**a**) Thermal maps showing the temperature variations (in °C) across the plantar surfaces of the feet, generated from interpolated insole sensor data. Temperature contours are superimposed on the thermal map, while the white circles in the background correspond to the sensors depicted in Figure 2. (**b**) The corresponding thermogram.

Figure 4a shows example thermal maps generated for one of the participants using the Clough–Tocher interpolator, while Figure 4b shows the corresponding thermogram captured with the FLIR E95 thermal camera—note the similarity of the thermal patterns, thus validating the interpolation method selected in this study.

### 2.4. Data Clustering

In contrast with existing work in the literature, we adopt a purely data-driven clustering approach to analyse normative foot thermal patterns in participants lying in a supine posture with shod feet equipped with smart insoles. This approach is not conditioned on a predetermined model, allowing patterns to emerge naturally from the data without the constraints of imposed spatial segmentation or anatomical assumptions.

Using the temperature data expressed as relative temperature differences previously described, we applied several clustering algorithms to explore and validate potential patterns in our data, selecting each method for its unique strengths: k-means, fuzzy c-means, the OPTICS algorithm (ordering points to identify the clustering structure), and hierarchical clustering. Each algorithm makes different assumptions about cluster shape and noise—for example, k-means assumes spherical clusters, while OPTICS can detect arbitrarily shaped clusters and handle noise. Hierarchical clustering, in particular, provides insights into the nested structure of clusters, allowing us to observe relationships between clusters at different levels of similarity. This diversity in clustering approaches allowed us to test for consistency and reliability across methods.

Due to the high dimensionality of the input data (21 sensors per foot, resulting in 21 dimensions), we performed dimensionality reduction via principal component analysis (PCA), in order to mitigate potential issues during clustering. This step reduced the data to 10 dimensions while retaining 98% of the explained variance.

In each clustering experiment, the left and right feet were considered separately from each other. Thus clustering was performed on n=54 observations, 2 observations (one for the left and the right legs) for each of the 27 participants. All clustering experiments were conducted using the Scikit-Learn library [33]. The Matplotlib [34] and Seaborn [35] libraries were employed for generating the clustering results visualizations.

### 2.5. Hyperparameter Selection

Where possible, clustering hyperparameters were selected automatically rather than manually. For the k-means algorithm, the optimal number of clusters, *k*, was determined using the elbow method, with the Kneedle algorithm [36] used to identify the elbow point in the inertia-versus-*k* plot. For fuzzy c-means, the number of clusters, *c*, was chosen by maximizing the fuzzy partition coefficient (FPC), which reflects the clarity and distinctness of the clusters [37]. For hierarchical clustering, agglomerative clustering with Ward linkage was manually selected. Finally, the OPTICS algorithm [38] required minimal parameter tuning, needing only a minimum number of samples per cluster to be specified—this was chosen empirically.

## 3. Results

**Figure 5:**
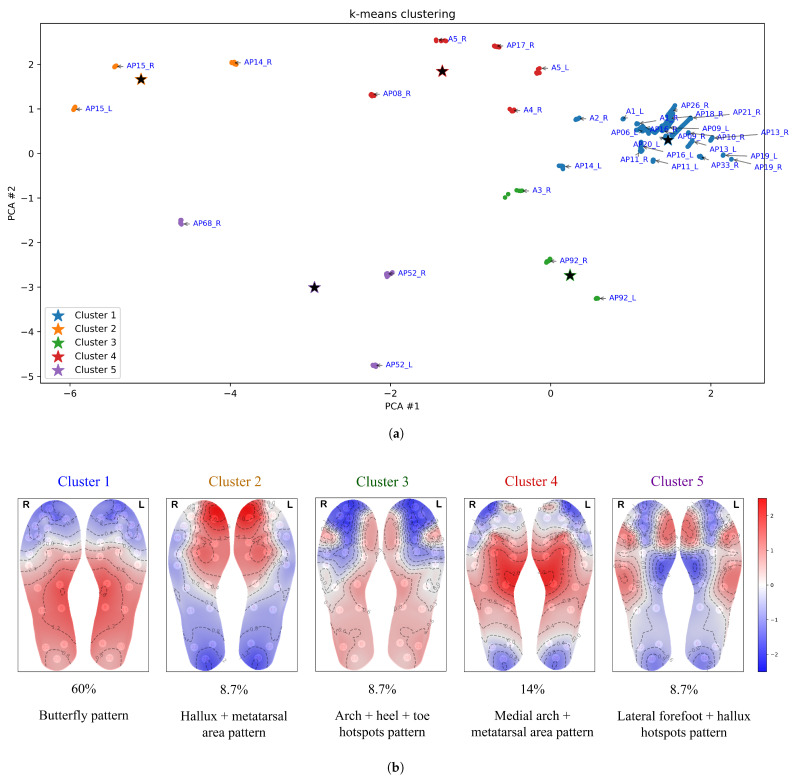
(**a**) Clustering results from the k-means algorithm with k=5. Cluster centroids are denoted by a star symbol. (**b**) Thermal maps of the cluster centroids, cluster sizes (%), and thermal pattern names.

**Figure 6:**
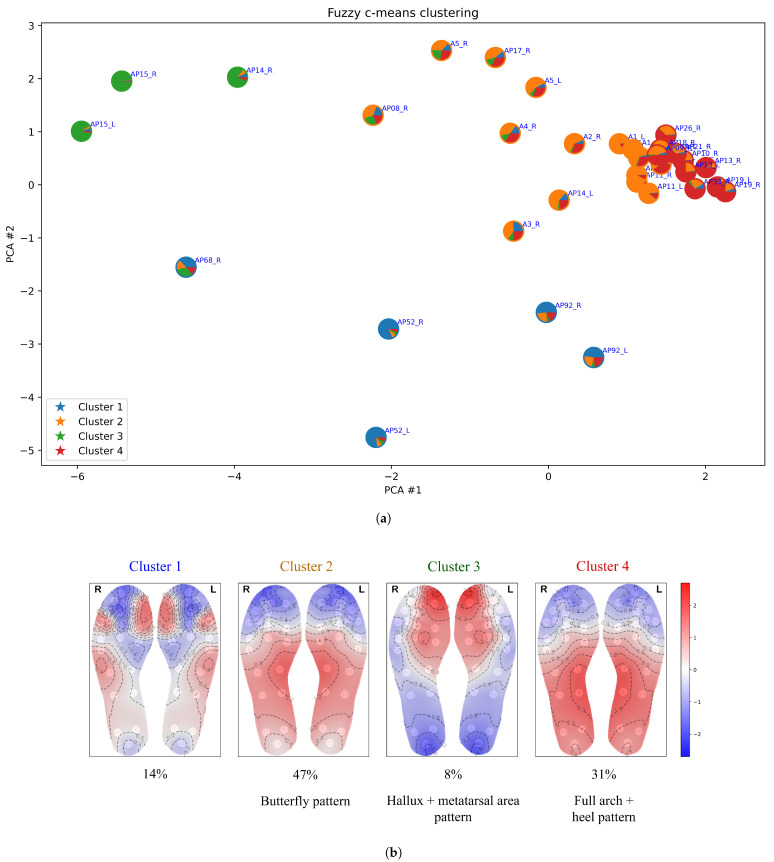
(**a**) Clustering results from the fuzzy c-means algorithm with c=4. Each data point is displayed as a pie chart, showing fuzzy membership across the five clusters, with the border colour indicating the final cluster assignment. (**b**) Thermal maps of the cluster centroids, cluster sizes (%), and thermal pattern names.

**Figure 7:**
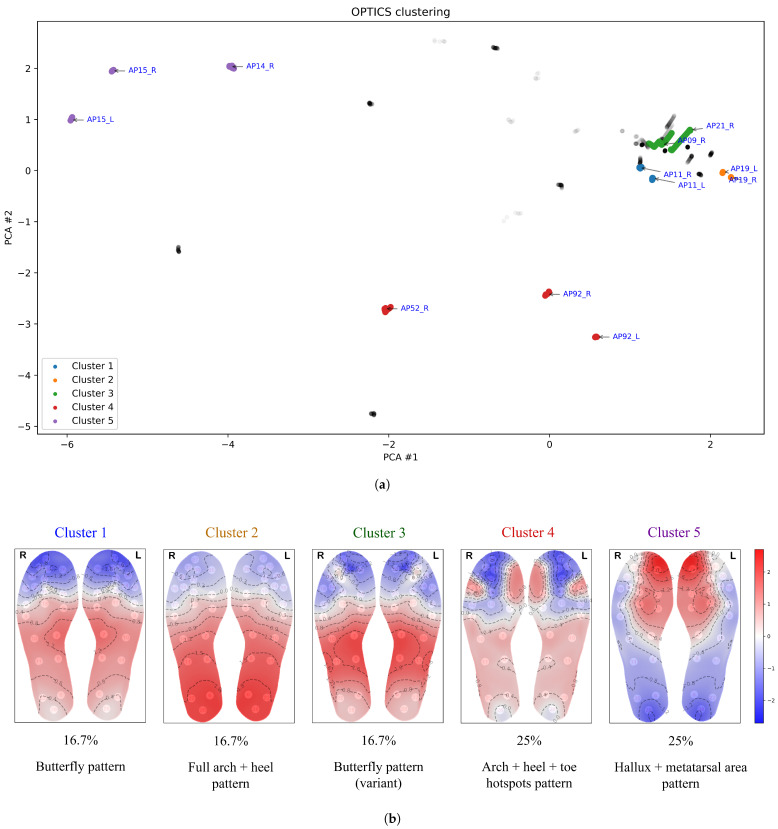
(**a**) Clustering results from the OPTICS algorithm, with outlier data points coloured grey. (**b**) Thermal maps of the cluster centroids, cluster sizes (%), and thermal pattern names.

**Figure 8:**
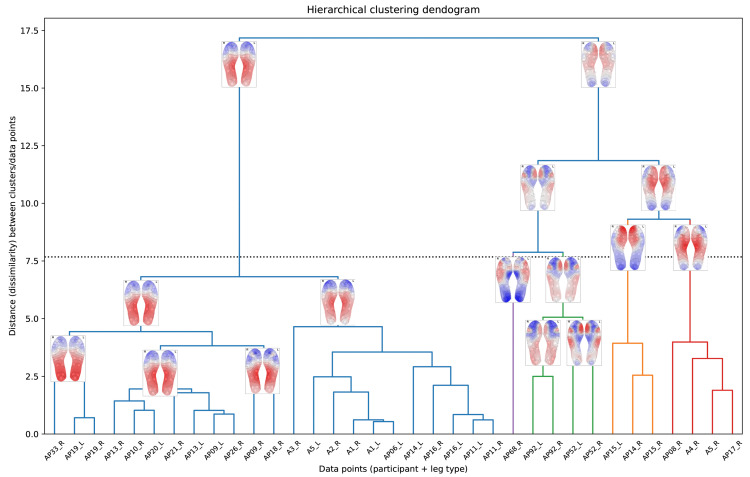
Dendogram representing the results of the hierarchical clustering algorithm. The horizontal dotted line corresponds to the cut-off threshold selected for the five-cluster results shown in Figure 9. The sub-trees below the cut-off are colour-coded to match the cluster colours used in Figure 9.

**Figure 9:**
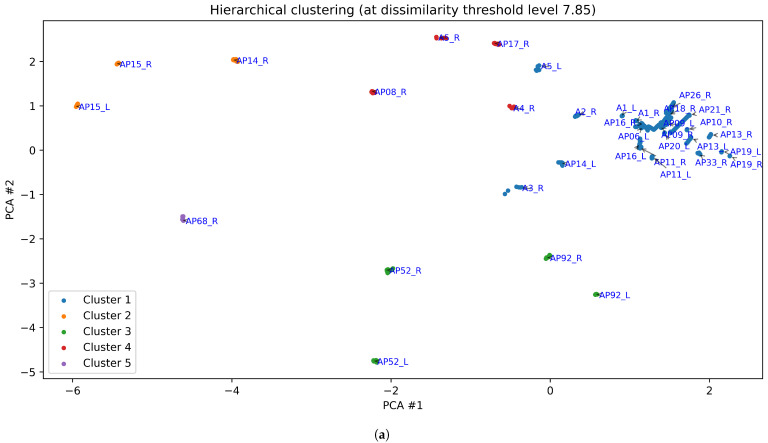

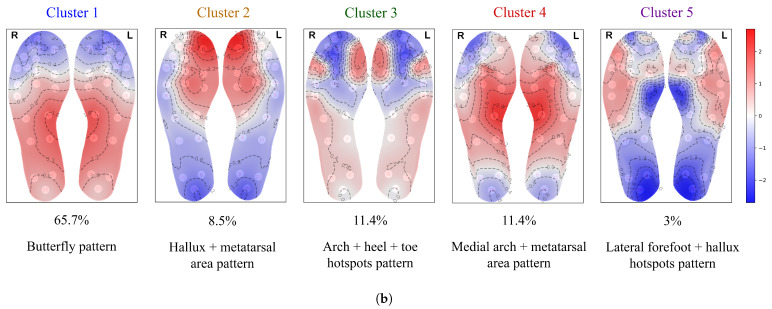
(**a**) Clustering results obtained with the hierarchical clustering algorithm, when setting the threshold level of dissimilarity at 7.85 (depicted as a horizontal dotted line in the dendogram of Figure 8). (**b**) Thermal maps of the cluster centroids, cluster sizes (%), and thermal pattern names.

Figure 5, Figure 6, Figure 7, Figure 8 and Figure 9 display the clustering results of the different clustering algorithms used in this study. The first of these figures, Figure 5a, shows a 2D plot of the clustering results of the k-means algorithm. The labels next to the data points indicate the participant identifier followed by the leg type indicator (L/R). Figure 5b shows the thermal maps corresponding to the cluster centroids, the cluster sizes (shown as a percentage of total data points), and the name of the thermal pattern (cluster) as referred to in this study. The thermal maps of the cluster centroids show the relative temperature difference (in °C) from the mean foot temperature (computed as the mean of the 21 sensors of each insole)—red areas in the thermal map indicate an elevated temperature compared to the mean, while blue areas indicate lower temperatures. A similar layout and structure is adopted for the remaining figures, Figure 6, Figure 7, Figure 8 and Figure 9.

Both k-means and OPTICS identified five clusters, while fuzzy c-means produced four clusters. Hierarchical clustering, which does not inherently define a specific number of clusters, was adjusted to five clusters (for the purpose of comparison) by selecting a threshold on the dendogram (see Figure 8), resulting in the five-cluster structure shown in Figure 9.

The largest cluster identified by the k-means algorithm (cluster 1 in Figure 5) exhibits the classical butterfly pattern, characterized by the medial arch as the warmest region, as reported in the literature [22,23,24,25]. This cluster is also the most concentrated, as shown in the figure. The second-largest cluster (cluster 4 in Figure 5) bears some resemblance to the butterfly pattern, but with the warmest areas extending further up toward the ball of the foot (metatarsal area) and toes. We refer to this thermal pattern as the “medial arch + metatarsal area” pattern. In the thermal pattern of cluster 2, the warmest areas are located at the hallux and the ball of the foot. Clusters 3 and 5 display thermal patterns with multiple hotspots. Cluster 5 shows an elevated temperature along the lateral edges of the forefoot and at the hallux, while cluster 3 exhibits less prominent temperature increase more broadly spread over the arch and heel region, with hotspots at the hallux and the lateral toe area.

Similarly, the largest cluster from fuzzy c-means (cluster 2 in Figure 6) also represents the classic butterfly pattern. However, fuzzy c-means splits some data points from the butterfly cluster into a separate cluster (cluster 4), which features elevated temperature in the heel region and a broader distribution across the midfoot and heel rather than concentrating solely on the medial arch—hence, we refer to it as the “full arch + heel” pattern. Additionally, cluster 1 of fuzzy c-means corresponds to a combination of clusters 3 and 5 from k-means, while cluster 3 aligns with cluster 2 from k-means. One characteristic of fuzzy c-means clustering is its ability to allow data points to belong to multiple clusters—this fuzzy membership is illustrated in terms of piecharts in Figure 6a. This is useful for analysing patterns that exhibit gradual transitions or overlap between them; for example, clusters 2 and 4 in Figure 6.

Figure 7 shows that the OPTICS clustering algorithm classifies many data points as outliers, assigning them to no cluster (displayed in grey in Figure 7). This outcome reflects the way OPTICS operates: it is designed to detect clusters with varying densities, where *density*, in the context of clustering, refers to how closely packed the data points are in a given area, with the proximity between points reflecting the similarity between temperature distributions. Points that are not densely connected to others within a specified distance threshold are treated as outliers. This is especially common in datasets with regions of differing densities or areas with low data density. Thus OPTICS distinguishes itself by identifying outliers (thermal patterns that are considerably distinct from others) rather than forcing these data points into clusters, making it particularly useful for detecting anomalies or isolated thermal patterns.

Clusters 1 and 3 from OPTICS both display characteristics of the classical butterfly pattern—an increase in temperature concentrated in the medial arch, with cooler areas in the forefoot and heel. However, OPTICS clustered them separately, likely due to its sensitivity to density-based variations. Minor differences in intensity or distribution within the medial arch and adjacent regions may have caused the algorithm to recognize them as distinct clusters. Cluster 2 from OPTICS corresponds to cluster 4 from fuzzy c-means, while clusters 4 and 5 from OPTICS align with clusters 3 and 2 from k-means, respectively.

Figure 9 shows the clustering results from hierarchical clustering, with a cut-off level selected to yield 5 clusters. Clusters 1 to 4 happen to correspond to the same thermal patterns as in k-means, while cluster 5 is similar to cluster 5 from k-means, except that the hallux shows a lower temperature in the hierarchical clustering results.

The dendogram in Figure 8 visually represents the hierarchical clustering process as a tree-like structure, enabling us to explore relationships between clusters and their thermal patterns, as well as observe how clusters are progressively combined into larger, more cohesive groupings. At the bottom of Figure 8 we observe the individual data points. Traversing the vertical axis of the dendogram in an upward direction, we observe the successive merging of the data points into clusters, and these clusters merging into larger clusters, finishing off with a single cluster at the top. Thermal maps are included in Figure 8, superimposed on the branches of the dendogram, and representing the ‘centroid’ or cluster centre of the cluster belonging to that branch.

This dendogram is useful as it offers insights into the nested structure of the clusters, enabling the exploration of subgroup relationships and the identification of patterns at varying levels of similarity. For example, clusters 2 and 4 (the sub-trees depicted in orange and red, respectively, in Figure 8) show a strong similarity between them even though the highest temperature occurs in different foot regions.

The dendogram also reveals that the butterfly pattern cluster further sub-divides into two related patterns: the classic butterfly pattern, and the “full arch + heel pattern” (observed in cluster 2 of OPTICS and cluster 4 of fuzzy c-means). This indicates that these two clusters share common characteristics in their thermal patterns. Additionally, the dendrogram highlights a variant of the butterfly pattern with a slight hotspot in the toe area, aligning with and explaining the results from the OPTICS algorithm (cluster 3 of Figure 7).

The horizontal dotted line in Figure 8 corresponds to the selected cut-off threshold (y≈7.85) for the clustering results shown in Figure 9, yielding five clusters at this level. The sub-trees below this cut-off are colour-coded to match the corresponding cluster colours used in Figure 9.

Across all clustering algorithms, the butterfly pattern, representing half of the dataset, is the most prominent and consistently identified thermal pattern, though OPTICS separates it into two related clusters (clusters 1 and 3) due to density sensitivity. The second most prominent pattern, the “full arch + heel” pattern, accounting for about a quarter of the dataset, also demonstrates good consistency, appearing as cluster 4 in both fuzzy c-means and OPTICS, while hierarchical clustering identifies it at a lower cut-off threshold. The remaining clusters, though less prevalent, show moderate to good consistency across methods, with minor variations stemming from differences in clustering approaches, underscoring their overall stability.

## 4. Discussion

### 4.1. Thermal Patterns

When comparing the results of the different clustering algorithms, we observe a high degree of similarity among them. Variations are primarily due to clustering granularity—whether coarse or fine-grained clusters are produced—which depends on the choice of the number of clusters (as in k-means, and fuzzy c-means) or the cut-off level selected in hierarchical clustering.

Additional differences stem from how each algorithm handles sparse data points (points far apart from each other) and low-density areas (regions with few data points). For example, k-means aims to find the most suitable cluster for each data point by minimizing the variance within clusters but may struggle with irregular cluster shapes. Fuzzy c-means allows data points to belong to multiple clusters, providing membership levels for each cluster, which can be useful for analysing thermal patterns that exhibit gradual transitions. Hierarchical clustering, on the other hand, offers insights into the nested structure of clusters of thermal patterns, enabling the identification of patterns at varying levels of similarity. OPTICS distinguishes itself by identifying outliers (thermal patterns that are considerably distinct from others), making it particularly useful for detecting anomalous patterns.

Table 1 summarizes the common thermal patterns identified by the clustering algorithms used in this study. The predominant cluster produced by all algorithms is the butterfly pattern. On average, across the four clustering methods, the butterfly pattern accounts for 51.5% of all data points—this is consistent with literature on thermograms of unshod feet, which report a range of 44% to 56% [22,23,24,25,26]. This suggests that the normative thermal patterns observed in thermograms of unshod feet are also present when using wearable insoles on shod feet.

We observe that the boundaries between clusters are not sharply defined, leading to potential inter-class intersection/overlap for both clustering and classification algorithms. Previous studies on thermal pattern clustering in thermograms [24,25] may have simplified the problem by using binary labelling for temperature differences and adopting a predetermined model for foot area segmentation, potentially obscuring these gradual transitions and the resulting cluster overlap.

### 4.2. Limitations

This study has a number of limitations. The small number of participants may influence the clustering results, potentially introducing bias and issues related to data sparsity. Although we took care to ensure proper contact between the feet and temperature sensors and to align the insoles accurately with the plantar surface, some variations may still have occurred, potentially impacting the clustering results and their analysis. To mitigate misalignment issues, we used different-sized insoles according to participant foot size, and comparing thermograms with the insole heatmaps gives us confidence that these variations have been minimized as much as possible (refer to Figure 4 for an example).

As mentioned earlier, the participants’ pose was restricted to a supine position, since the focus of this study was to establish baseline thermal patterns in shod feet while under conditions similar to those typically used in unshod thermographic studies. Moreover, this study was limited to normative thermal patterns and did not attempt to infer any conclusions about the participants’ actual health conditions based on the thermal pattern clustering results.

## 5. Conclusions

This study is the first to apply data-driven clustering to temperature data from wearable insole-based sensors, uncovering normative thermal patterns in shod feet. By employing multiple clustering algorithms—k-means, fuzzy c-means, OPTICS, and hierarchical clustering—we identified several common thermal patterns, with the butterfly pattern consistently emerging as the predominant cluster across methods. The thermal patterns identified in this study align well with the normative thermal patterns reported elsewhere in the literature for unshod thermographic imaging. This highlights the potential of insole-based systems for continuous foot health monitoring in real-world conditions.

Such wearable insole-based systems offer the unique advantage of monitoring inside the shoe and on the go, providing a more realistic representation of physiological activity and foot health during daily activities. Such systems can gather objective data over extended periods of time, unlike traditional thermography, which captures only limited snapshots of unshod feet. And, with the integration of mobile devices, these systems become both feasible and convenient. Data can be seamlessly transferred via Bluetooth from the insole to a mobile device, and subsequently to the cloud for long-term storage and access by clinicians.

Future work will extend this approach to other static postures, as well as to dynamic activities such as walking, which would not be possible with conventional thermal imaging. Additionally, we aim to extend this work to analyse differences in thermal patterns between healthy individuals and those with diabetes.

## Figures and Tables

**Table 1:** Common thermal patterns identified by the clustering algorithms.

Thermal Pattern/Clustering Algorithm	Butterfly Pattern	Full Arch + Heel	Hallux + Metatarsal Area	Medial Arch + Metatarsal Area	Arch + Heel + Toe Hotspots	Lateral Forefoot + Hallux Hotspots
k-means	Cluster 1		Cluster 2	Cluster 4	Cluster 3	Cluster 5
Fuzzy c-means	Cluster 2	Cluster 4	Cluster 3		Cluster 1	Cluster 1
OPTICS	Clusters 1, 3	Cluster 2	Cluster 5		Cluster 4	
Hierarchical clustering	Cluster 1	*	Cluster 2	Cluster 4	Cluster 3	Cluster 5
Thermal pattern heatmap and % of occurrence averaged across clustering methods	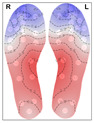	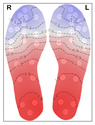	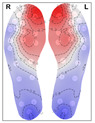	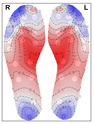	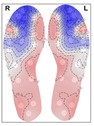	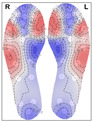
	51.5%	23.8%	12.6%	12.6%	14.8%	8.6%

* This pattern is observed at a lower cut-off level for hierarchical clustering (see Figure 8).

## Data Availability

The data used in this study are not publicly available due to privacy and ethical restrictions, as they contain sensitive patient information. Access to the data is restricted to comply with confidentiality agreements and institutional regulations.
